# LPS Regulates Endometrial Immune Homeostasis and Receptivity Through the TLR4/ERK Pathway in Sheep

**DOI:** 10.3390/ani15121712

**Published:** 2025-06-10

**Authors:** Jinzi Wei, Xing Fan, Xiaorui Zang, Yu Guo, Wenjie Jiang, Meiyu Qi, Hongbing Han, Yuchang Yao

**Affiliations:** 1 College of Animal Science and Technology, Northeast Agricultural University, Harbin 150030, China; 13793309337@163.com (J.W.); fstar0213@163.com (X.F.); zangxr8683@163.com (X.Z.); jiegeng2023@163.com (Y.G.); jwj@neau.edu.cn (W.J.); 2 Institute of Animal Husbandry, Heilongjiang Academy of Agricultural Sciences, Harbin 150028, China; joan7843@163.com; 3 College of Animal Science and Technology, China Agricultural University, Beijing 100193, China

**Keywords:** embryo implantation, inflammation, endometrial receptivity, TLR4

## Abstract

In sheep production, due to the limitations of breeding conditions, the uteri of ewes are often infected with bacteria, resulting in the failure of embryo implantation or loss, causing huge losses to the sheep industry. Therefore, in this study, by using RT-qPCR, Western blot, and immunofluorescence, we investigated the effects of LPS infusion on the immune microenvironment and endometrial receptivity, which play an important role in the process of embryo implantation in ruminants, during the three critical periods of embryo implantation in sheep. The results showed that LPS infusion at day 12, day 16, and day 20 significantly increased the expression of Th1 cytokines (TNF-α, IL-1β, IL-8, IL-6), while significantly decreasing the expression of Th2 cytokines (IL-4 and IL-10) and disrupting the expression of implantation factors, such as *ITGB3*, *ITGB5*, *VEGF*, and *LIF*, in the endometrial tissues of sheep. Additionally, the protein expression level of TLR4 and the phosphorylation level of ERK were significantly elevated at day 12, day 16, and day 20 after LPS infusion, suggesting that LPS may impair endometrial receptivity through the TLR4/ERK pathway. Validation was conducted in a receptive model of sEECs using TLR4 and ERK phosphorylation inhibitors. Compared with the LPS group, TLR4 and ERK phosphorylation inhibitors significantly reduced the expression of TLR4 and p-ERK, down-regulated Th1 cytokines, up-regulated Th2 cytokines, and alleviated the disruption of genes for attachment. Treatment with 50 μM PTE can significantly alleviate the abnormal expression of implantation genes caused by LPS, and its mechanism may be related to the regulation of the ERK signaling pathway.

## Simple Summary

LPS, present in the outer membrane of Gram-negative bacteria, acts as a miscarriage-causing effector that impairs the normal physiological function of the endometrium, resulting in its inability to successfully accept embryos for implantation. However, the mechanism of LPS damage in ruminants remains unelucidated. In this study, we investigated the expression patterns of Th1/Th2 immune balance and attachment-related genes in endometrial tissues during the embryo attachment stage and the effects of LPS exposure on them, further elucidating the molecular mechanisms of LPS damage to endometrial epithelial cells in sheep, and demonstrated the mitigating effect of pterostilbene. The results showed that LPS disrupted the immune balance by activating TLR4 and its downstream ERK pathway, leading to a shift of in utero immune homeostasis toward Th1, which is unfavorable to embryo implantation and interfered with the normal expression of adhesion genes and implantation marker genes. In vitro experiments showed that the addition of pterostilbene attenuated the damage caused by LPS. These findings enrich the knowledge of the mechanisms of gestational immune receptivity in non-invasive embryonic pregnant species and may provide new insights into therapeutic targets for LPS-induced endometrial cell injury in sheep.

## 1. Introduction

Implantation failure is a major obstacle to the establishment of ruminant gestation which affects the improvement of ruminant fertility, causing economic losses [1]. Synchronous communication between the developing embryo and the receptive endometrium is key to embryo implantation, which in the case of the uterus, provides the embryo with the opportunity to attach, invade, and develop. It is affected by a variety of factors, such as hormone levels, immune regulation, and various cytokine expressions [2].

In sheep, after hatching from the zona pellucida, the blastocyst expands into an ovoid and is termed a conceptus [3]. The ovoid conceptus begins to elongate on day 12 of early pregnancy. The elongation of the conceptus marks the beginning of implantation and then elongates to a filamentous conceptus between days 12 and 15 [4]. After conceptus elongation, the conceptus begins to differentiate into binucleate trophoblast cells (BNCs) [5]. The BNCs are inherently invasive, with weak attachment to the endometrium occurring at approximately day 16 [6]. And central implantation and placentation of the elongated conceptus begin on day 16 in sheep [4]. At this time, Th1 immunity, characterized by immune-inflammatory responses, becomes dominant and “controlled” Th1 immunity benefits the invading trophoblasts rather than harming them [7]. On day 20, the trophoblasts adhere firmly to the endometrial epithelium. The implantation is complete [8]. Quickly, early inflammatory Th1 immunity is shifted to the Th2 anti-inflammatory immune responses. The predominant Th2 immunity, which overrules Th1 immunity at the placental implantation site, protects a fetus by balancing Th1 immunity and accommodating fetal and placental development [9]. Although there is a brief Th1 deviation during implantation, the embryo expresses a partial paternal alloantigen, considered a semi-allogeneic graft (semi-allograft) that has been transferred to the mother [10]. In order to prevent rejection of the semi-allogeneic fetus, the maternal immune system regulates various immune cells and promotes the transfer of cytokine balance to anti-inflammatory Th2 immunity, thereby promoting maternal and fetal tolerance [11,12].

Toll-like receptor 4 (TLR4) is broadly expressed at the maternal–fetal interface [13]. Previous studies have shown that TLR4 plays an important role in mouse and porcine embryo implantation [14,15]. TLR 4 is an essential member of the TLR family. TLR4 binding to lipopolysaccharide (LPS) activates two distinct signaling pathways: the myeloid differentiation primary response 88 (MyD88) pathway and the Toll/IL-1R domain-containing adapter-inducing IFN-β (TRIF) pathway [16]. Both pathways ultimately activate the NF-kB and MAPK pathways [17]. TLR4 regulates the production of inflammatory cytokines via the NF-κB and MAPK pathways, such as inflammatory cytokines, interleukins, tumor necrosis factors, and prostaglandins, which affect the inflammatory microenvironment of the uterus [18]. Moreover, a prerequisite for adhesion and migration of trophectoderm is activation of mTOR, PI3K, MAPK (ERK1/2), and MAPK14 (p38) signaling [19]. In chronic endometriosis and chronic endometritis, the expression of the MAPK pathway is abnormally elevated, affecting the function of endometrial cells [20,21,22].

Bacterial infections of the reproductive tract caused by Gram-negative bacteria, like *Escherichia coli*, can lead to infertility and early pregnancy failure. Lipopolysaccharide (LPS) is a major component of the cell wall of Gram-negative bacteria [23]. Exposure of pregnant rats to bacterial LPS resulted in maternal inflammations and fetal loss, as well as structural abnormalities in the uteroplacental vasculature [20]. LPS induced DNA damage in the preimplantation-stage embryos and uterine cells, which may ultimately inhibit the process of implantation in mice [24]. LPS induce chronic inflammatory processes affecting the endometrium, as encountered in endometriosis, adenomyosis, chronic endometritis, and obstruction of normal implantation [25].

Therefore, we hypothesized that intrauterine infusion of LPS would disrupt the expression patterns of Th1/Th2 immune balance and implantation-related genes in endometrial tissues during the embryo attachment stage and be detrimental to the process of implantation. In this study, we investigated the effects and mechanisms of LPS on embryo implantation failure based on the TLR4/ERK pathway by focusing on implantation-related genes and Th1-TH2 cytokine expression in sheep at different stages of implantation and in an in vitro cell model, moreover demonstrating the alleviating effect of PTE on the damage caused by LPS.

## 2. Materials and Methods

### 2.1. Ethics Statement

All animal experiments and treatments followed the guidelines of the Animal Welfare Committee of the Northeast Agricultural University, and all experiments were approved by the Animal Welfare Committee of the Northeast Agricultural University (experimental license: NEAUEC20210207).

### 2.2. Collection of Uterine Tissues

Healthy and nulliparous 12-month-old German Mutton Merino sheep with normal estrous cycles were selected for this study. Thirty-five ewes were randomly divided into seven groups (*n* = 5 for each group). Considering that some ewes may fail to conceive, 49 ewes were actually used, with 2 more added to each group. All ewes were synchronized with estrus using a controlled vaginal release device (CIDR) buried for 14 days and simultaneously intramuscularly injected with 0.1 mg PGF_2α_ and 330 IU pregnant mare serum. Starting from the 36th hour post-withdrawal of the CIDR intravaginal device, estrus detection was performed every 8 h using a ram. Semen of Merino sheep collected artificially was diluted with sterile dilution fluid (prepared with endotoxin-free water) to a concentration of 2.5 × 10^8^/mL; the semen collection method and sterile dilution fluid preparation were as described in our previous reports [26]. At 12 h after the ewe entered estrus, deep intrauterine artificial insemination into the ovulatory uterine horn was performed using a laparoscope, with 0.1 mL of diluted semen being inputted into the ovulatory uterine horn. The specific time points of animal tissue collection were as described in our previous reports [27]. Four sampling time points were selected according to the different stages of sheep embryo implantation: day 0: zygote formation; day 12: conceptus elongation; day 16: establishment of cell links between the embryo and the uterine epithelium; day 20: implantation completion. Twelve hours after artificial insemination was recorded as day 0, and uterine tissue was collected as the day 0 group. At sampling time points day 12, day 16, and day 20, a 1 mg/mL LPS (*Escherichia coli O111:B4*) concentrate was first configured, followed by dilution of 80 µL of LPS concentrate with 1.52 mL of phosphate-buffered saline (PBS). At each designated sampling node (day 12, day 16, and day 20), 10 individuals were allocated, and 0.8 mL LPS (treatment groups) was perfused into the uterus of 5 randomly selected individuals via laparoscopy combined with a uterine horn insemination gun, 24 h before sampling on day 11, day 15, and day 19. The remaining 5 individuals at each time point received the same amount of PBS (control groups) through the same procedure. After anesthetizing the ewe, the entire uterus was removed and longitudinally sliced under sterile conditions. After the longitudinal incision was made in the uterus, successful pregnancy was first confirmed (embryos appeared on days 12, 16, and 20). Then, the uterus was flushed with PBS and maternal endometrial tissue (1 mm thickness) samples were taken at the maternal–fetal interface (the uterine body including uterine caruncles). The tissues were immediately transferred to a 2 mL DNase/RNase-free tube filled with RNAlater (Qiagen, Valencia, CA, USA) and stored at −80 °C.

### 2.3. Cell Culture and Drug Treatment

The sheep endometrial epithelial cells (sEECs) were obtained from the laboratory of the College of Animal Science and Technology, China Agricultural University, provided kindly by Hongbing Han. The sEECs were seeded in a dish containing Dulbecco’s modified eagle medium (DMEM) high glucose supplemented with 10% fetal bovine serum (Pricella Biotechnology Co., Ltd., Wuhan, China.) and 1% Pen-Strep (10,000 U/mL penicillin and 10 mg/mL streptomycin (Biosharp Biotechnology Co., Ltd., Hefei, China) and incubated at 37 °C in a humidified 5% CO_2_ incubator. The medium was replaced with fresh medium every day. The sEECs were seeded in six-well plates, and upon reaching a cell density of 70–80%, the following treatments were initiated: (1) the inflammation cell model: sEECs were treated with 0, 1, 5, 10, and 20 µg/mL LPS (L4391; Sigma-Aldrich, St. Louis, MO, USA) for 6, 12, and 24 h; (2) The receptive cell model: the sEECs were cultured in fresh high-glucose DMEM with 0.1% bovine serum albumin (BSA) for 12 h [27,28]. Then, P_4_ (10^−7^ M, Sigma-Aldrich, St. Louis, MO, USA) and E_2_ (10^−9^ M, Sigma-Aldrich, St. Louis, MO, USA) were added to the medium. After hormone treatment for 12 h, the sEECs were treated with 20 ng/mL recombinant ovine IFN-τ (C600063; Sangon Biotech, Shanghai, China) for 12 h. Then, 5 µg/mL LPS (L4391; Sigma-Aldrich) was added to the medium. (3) Inhibitor treatment: in the presence of TAK242 (CLI095, Invitrogen, Carlsbad, CA, USA) and PD98059 (P215, Cell Signaling Technology, Danvers, MA, USA), 1 µM TAK242 and 10 µM PD98059 were added to the EECs before adding LPS for 1.5 h. The basis for choosing the concentration of TLR4 and ERK inhibitor was according to the studies of Gao et al. (2023) [29] and Hoon Kyu Oh et al. (2012) [30]. (4) The addition of PTE: 10, 50, and 100 µM PTE (HY-N0828, MedChemExpress, Monmouth Junction, NJ, USA) were added to the EECs after adding IFN-τ for 12 h and 24 h to determine the appropriate time and concentration. PTE was cotreated with LPS for 12 h in subsequent experiments.

### 2.4. CCK-8 Assay

sEECs were seeded in 96-well plates. After 24 h, sEECs in the wells were (1): treated with LPS (at 0, 1, 5, 10, and 20 μg/mL LPS) for 6 h, 12 h, and 24 h; (2): treated with PTE (at 0, 10, 50, and 100 μM PTE) for 12 h and 24 h; CCK8 (10 μL) was added to each well and incubated for 3 h. Finally, the optical density (OD) at 450 nm was measured with a microplate reader.

### 2.5. Western Blot

The cells were harvested and lysed using RIPA buffer (Beyotime Biotechnology, Shanghai, China) with a protease inhibitor cocktail and PMSF (Roche, Basel, Switzerland). Then, the proteins were quantified using the BCA Protein Assay Kit (Beyotime Biotechnology, Shanghai, China). Equal amounts of proteins were resolved on 10-12% SDS-PAGE and transferred to a polyvinylidene fluoride membrane (Millipore, Billerica, MA, USA). After incubation with primary antibodies against TLR4 (1:1000; AF7017; Afffnity Biosciences, Cincinnati, OH, USA), p-EKR (1:1000; Affinity, AF1015), β-actin (1:1000; Affinity, AF7018), and horseradish peroxidase-conjugated secondary antibodies (1:3000; Beyotime, A0208), the membranes were visualized by enhanced chemiluminescence (Cat No. 34577; Thermo Fisher Scientiffc, Cleveland, OH, USA). The protein bands were analyzed by ImageJ software (version 1.45; National Institutes of Health, Bethesda, MD, USA). Original Western Blot could be found as Supplementary Materials.

### 2.6. Quantitative Reverse-Transcription Polymerase Chain Reaction (qRT-PCR) Analysis

Total RNA was isolated with the Cell Total RNA Isolation Kit (Foregene, Chengdu, China) according to the manufacturer’s instructions, and ABclonal (RK20409, ABclonal Technology Co., Ltd., Wuhan, China) was used to generate cDNA (RNA:1000 ng). The mRNA expression levels were measured by qRT-PCR on a Roche LightCycler 480 instrument (Roche, Basel, Switzerland). GAPDH was chosen as the reference gene. All primer sequences are shown in Table 1. The 10 μL reaction system consisted of 5 µL of ROX (4913850001; Roche), 0.3 µL each of forward and reverse primers (0.3 μM), 1.0 μL of cDNA (1000 ng), and 3.4 μL of RNase-free water. The qRT-PCR procedure was as follows: pre-denaturation at 95 °C for 30 s; denaturation at 95 °C for 30 s, followed by 40 cycles of 95 °C for 10 s, 60 °C for 20 s, and 72 °C for 10 s. The results of mRNA expression were calculated by the 2^−ΔΔCT^ method. Details of the primer sequences are provided in Table 1.

### 2.7. Immunofluorescence Microscopy Analysis

The cells were washed with pre-cooled PBS and fixed with 4% paraformaldehyde for 15 min. Then, 0.5% Triton X-100 was used for cell permeabilization for 30 min. After that, the permeabilized cells were blocked with 2% BSA for 1 h and incubated with a primary antibody against cytokeratin 18 (1:200; AF0191; Affinity) at 4 °C overnight, followed by a fluorescence-labelled secondary antibody (1:200; A0516; Beyotime) at room temperature for 1 h. The nuclei were stained using DAPI (Beyotime, Haimen, China). The images were obtained using a fluorescence inversion microscope.

### 2.8. Statistical Analysis

All data are shown as the mean ± SEM, and individual experiments were repeated not fewer than three times. Statistical analyses were performed by the univariate analysis of variance (ANOVA), followed by Student’s *t*-test. *p* < 0.05 was considered to be statistically significant.

## 3. Results

### 3.1. Effect of LPS on the Expression Levels of Th1-Th2 Cytokines in the Endometrium of Sheep During Three Periods

**Figure 1:**
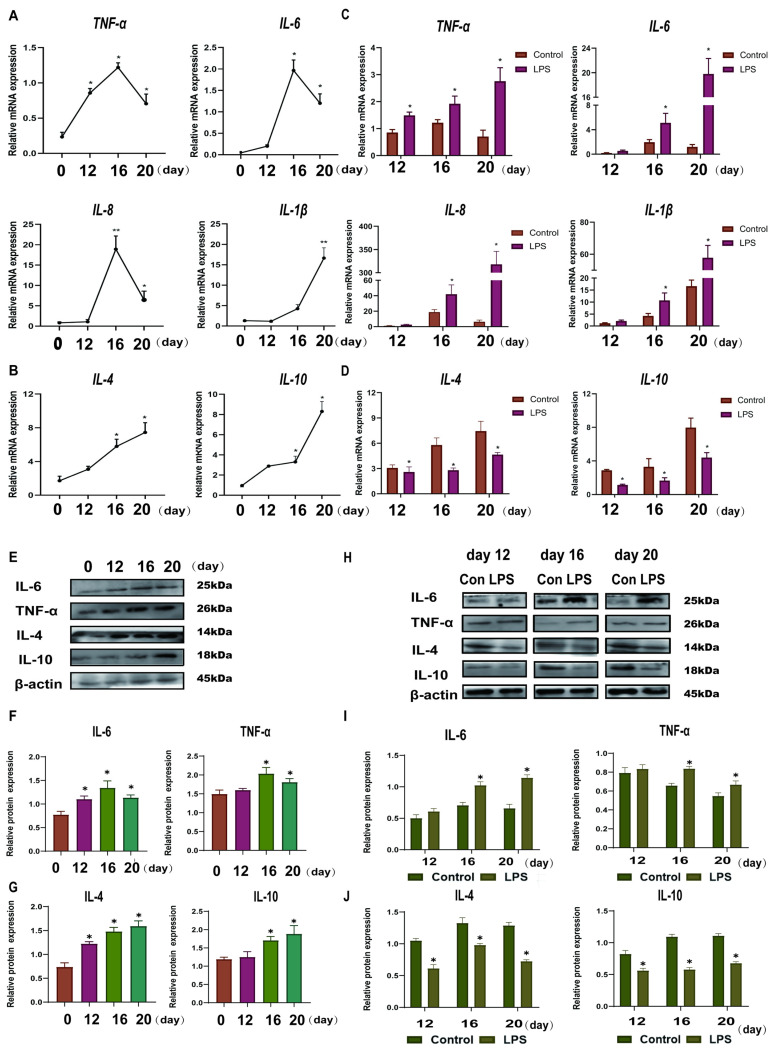
Effect of LPS on Th1-Th2 cytokines in the endometrium of sheep during three periods. (**A**): The mRNA expression of Th1 pro-inflammatory cytokines (*IL-1β*, *IL-8*, *TNF-a*, and *IL-6*) in the uterine tissue at days 0, 12, 16, and 20 of early pregnancy in sheep were detected by qPCR. (**B**): The mRNA expression of Th2 anti-inflammatory cytokines (*IL-10* and *IL-4*) in the uterine tissue at days 0, 12, 16, and 20 of early pregnancy in sheep were detected by qPCR. (**C**): The mRNA expression of Th1 pro-inflammatory cytokines (*IL-1β*, *IL-8*, *TNF-a*, and *IL-6*) in LPS-infused uterine tissue during three periods (days 12, 16, and 20) were detected by qPCR. “*” or “**” indicates that the treatment groups are different from control groups (and not between treatment groups). (**D**): The mRNA expression of Th2 anti-inflammatory cytokines (*IL-10* and *IL-4*) in LPS-infused uterine tissue during three periods (days 12, 16, and 20) were detected by qPCR. “*” indicates that the treatment groups are different from control groups (and not between treatment groups). (**E**–**G**): Expression of Th1 cell cytokine (TNF-a and IL-6) and Th2 cytokine (IL-10 and IL-4) proteins in uterine tissues analyzed by Western blot analysis. (**H**–**J**): Expression of Th1 cells cytokines (TNF-a and IL-6) and Th2 cytokines (IL-10 and IL-4) proteins in LPS-infused uterine tissues analyzed by Western blot analysis. “*” indicates that the treatment groups are different from control groups (and not between treatment groups). Data are represented as the mean ± SEM of three independent experiments. *, *p* < 0.05; **, *p* < 0.01.

To investigate the effect of LPS on the expression levels of Th1-Th2 cytokines in the endometrium, Th1 cytokines (TNF-a, IL-8, IL-6, and IL-1β) and Th2 cytokines (IL-10 and IL-4) were detected in endometrial tissues by qRT-PCR and WB. The Th1 cytokines, *TNF-a*, *IL-6*, and *IL-8* were found to increase in pregnancy from day 0 to day 16, peak on day 16, and then begin to decline until day 20 of pregnancy. However, *IL-1β* was gradually increased from day 0 to day 20 (*p* < 0.05; Figure 1A). The Th2 cytokines (*IL-10* and *IL-4*) gradually increased from day 0 to day 20 (*p* < 0.05; Figure 1B). The protein expression levels of IL-6, TNF-α, IL-4, and IL-10 were consistent with that at the gene levels (*p* < 0.05; Figure 1E–G). Th1 cytokines (*TNF-a*, *IL-8*, *IL-6*, and *IL-1β*) were significantly up-regulated, and Th2 cytokines (*IL-10* and *IL-4*) were significantly down-regulated after LPS infusion on days 12, 16, and 20 (*p* < 0.05; Figure 1C,D). There are congruence between protein level and gene level expression in IL-6, TNF-*a*, IL-4, and IL-10 (*p* < 0.05; Figure 1H–J). Based on the above results, LPS induces a shift in the immune micro-environment of sheep endometrial tissue towards the Th1 type.

### 3.2. Effect of LPS on the Expression of Endometrial Implantation Genes in Sheep at Three Periods

**Figure 2:**
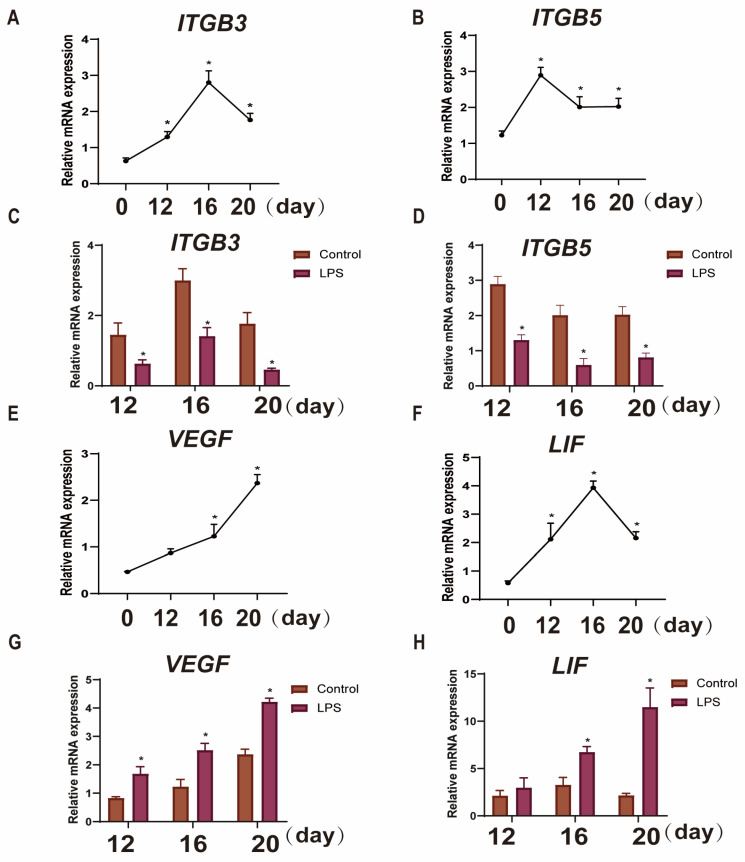
Effects of LPS on the expression of endometrial implantation genes in sheep during the three periods. (**A**,**B**): The mRNA expression of the cell adhesion genes *ITGB3* and *ITGB5* in uterine tissue at days 0, 12, 16, and 20 of early pregnancy in sheep were detected by qPCR. (**C**,**D**): The mRNA expression of the cell adhesion genes *ITGB3* and *ITGB5* in LPS-infused uterine tissue during three periods (days 12, 16, and 20) were detected by qPCR. “*” indicates that the treatment groups are different from control groups (and not between treatment groups). (**E**,**F**): The mRNA expression of the attachment indicators *VEGF* and *LIF* in the uterine tissue at days 0, 12, 16, and 20 of early pregnancy in sheep were detected by qPCR. (**G**,**H**): The mRNA expression of the attachment indicators *VEGF* and *LIF* in LPS-infused uterine tissue during three periods (days 12, 16, and 20) were detected by qPCR. “*” indicates that the treatment groups are different from control groups (and not between treatment groups). Data are represented as the mean ± SEM of three independent experiments. *, *p* < 0.05.

To examine the effect of LPS on endometrial implantation genes during the three critical periods of implantation in sheep, the mRNA expression levels of the adhesion genes (*ITGB3* and *ITGB5*) and the attachment genes (*VEGF* and *LIF*) in endometrial tissues were measured by qRT-PCR. The adhesion gene *ITGB3* was found to increase in pregnancy from day 0 to day 16, peak on day 16, and then begin to decline until day 20 of pregnancy (*p* < 0.05; Figure 2A). However, *ITGB5* increased from day 0 to day 12 and gradually decreased at day 16 and 20 (*p* < 0.05; Figure 2B). The attachment gene *VEGF* gradually increased from day 0 to day 20 (*p* < 0.05; Figure 2E). *LIF* increased during pregnancy from day 0 to day 16, peaked on day 16, and then began to decline until day 20 of pregnancy (*p* < 0.05; Figure 2F). The adhesion genes (*ITGB3* and *ITGB5*) were significantly down-regulated (*p* < 0.05; Figure 2C,D), and attachment genes (*VEGF* and *LIF*) were significantly up-regulated after LPS infusion on days 12, 16, and 20 (*p* < 0.05; Figure 2G,H).

### 3.3. Effect of LPS on the TLR4/ERK Pathway in Sheep Endometrium Tissues

**Figure 3:**
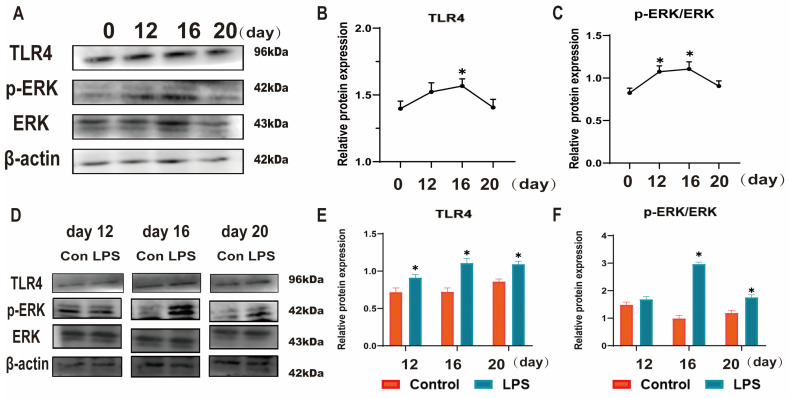
Effect of LPS on the TLR4/ERK pathway in sheep endometrium tissues. (**A**–**C**): The protein levels of TLR4, p-ERK and ERK in the uterine tissue at days 0, 12, 16, and 20 of early pregnancy in sheep were detected by Western blot. (**D**–**F**): The protein levels of TLR4, p-ERK, and ERK in LPS-infused uterine tissue during three periods (days 12, 16, and 20) were detected by Western blot. “*” indicates that the treatment groups are different from control groups (and not between treatment groups). Data are represented as the mean ± SEM of three independent experiments. *, *p* < 0.05.

TLR4 plays an important role in the establishment and maintenance of pregnancy and immunological reaction in animals, and ERK is one of the downstream molecules of TLR4. To reveal the mechanism by which LPS affects endometrial receptivity, we used Western blotting to analyze the TLR4 expression level and p-ERK/ERK ratio. Figure 3A–C shows that the TLR4 expression levels and p-ERK/ERK ratio increased from day 0 to day 16 and decreased from day 16 to day 20 (*p* < 0.05). The TLR4 expression levels and p-ERK/ERK ratio were significantly increased after LPS infusion at three time points (*p* < 0.05; Figure 3D–F). Based on the above, we hypothesized that LPS induces hyperactivation of the TLR4/ERK pathway in endometrial tissues of sheep during implantation.

### 3.4. Establishment of a Model of Endometrial Epithelial Cell Inflammation in Sheep

**Figure 4:**
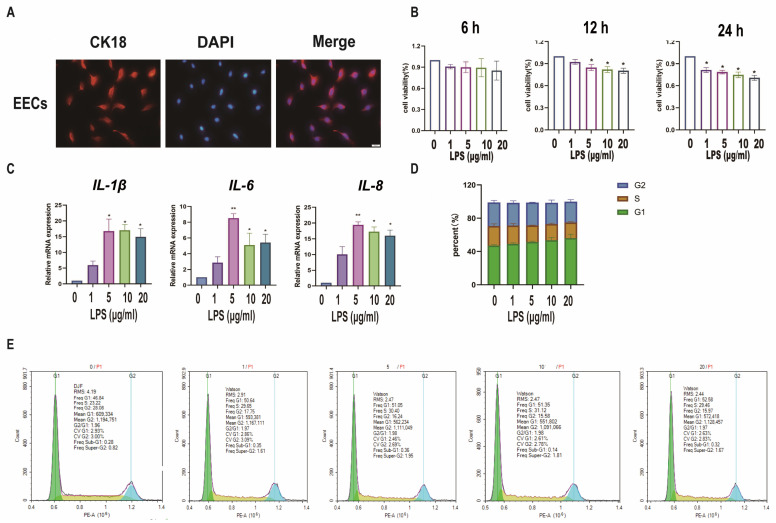
Establishment of a model of endometrial epithelial cell inflammation in sheep. (**A**): Detection of cell markers by immunofluorescence staining. EECs of sheep expressing CK18. Bar = 100 µm. (**B**): The cell viability of sEECs treated with LPS at five concentrations (0, 1, 5, 10, 20 μg/mL) for 6, 12, and 24 h by the CCK-8 assay. (**C**): The mRNA levels of *IL-6*, *IL-1β*, and *IL-8* were determined using qRT-PCR after sEECs were treated with 0, 1, 5, 10, and 15 μg/mL LPS for 12 h; (**D**,**E**): Effect of LPS on cell cycle regulation in sEECs, as assessed by flow cytometry of propidium iodide-stained cells; Data are represented as the mean ± SEM of three independent experiments. *, *p* < 0.05; **, *p* < 0.01.

For the hypothesis, sEECs were treated with LPS to mimic the inflammatory state. The specific marker protein CK18 was used to identify sEECs (Figure 4A). To determine the appropriate concentration of LPS, we treated sEECs with five concentrations of LPS (0, 1, 5, 10, 20 μg/mL LPS) and three exposure times (6 h, 12 h, 24 h) to assess the effect of LPS on cell proliferation, cell cycle, and inflammatory factors. The CCK-8 assay was applied to determine the effect of LPS on the viability of sEECs. As shown in Figure 4B, 5 μg/mL LPS treatment for 12 h significantly inhibited cell viability (*p* < 0.05). Moreover, 5 μg/mL LPS treatment for 12 h significantly increased IL-1β, IL-6, and IL-8 expression levels (*p* < 0.05; Figure 4C). Furthermore, flow cytometric analysis of the cell cycle showed that 5 μg/mL LPS caused G1 phase arrest (Figure 4D,E). Taken together, 5 μg/mL LPS treatment for 12 h was selected for further experiments.

### 3.5. LPS Affects Sheep Endometrium Through TLR4/ERK Pathway

**Figure 5:**
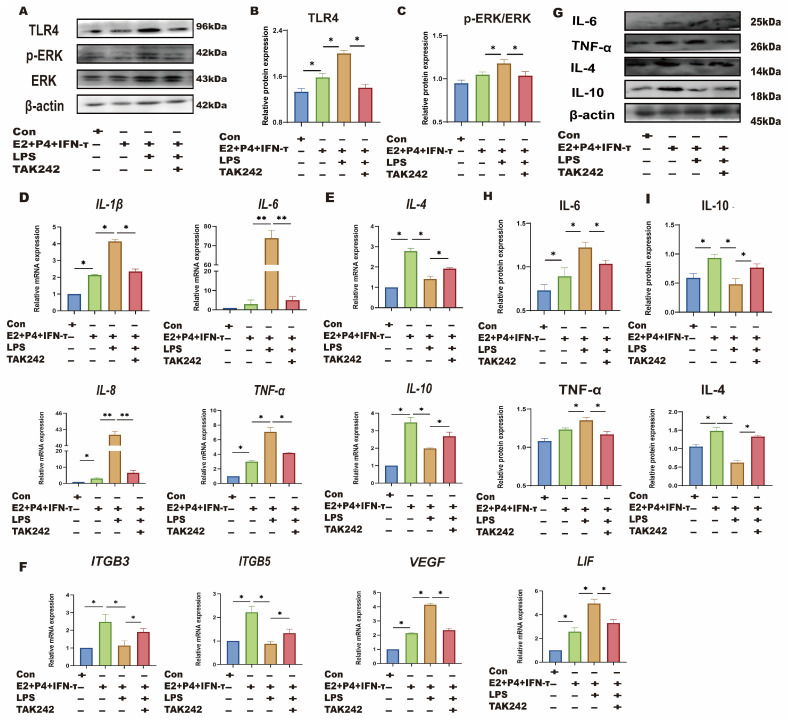
LPS affects endometrial receptivity in sheep through the TLR4 pathway. The sEECs were treated with 10^−7^ M P_4_, 10^−9^ M E_2_ for 12 h, 20 ng/mL IFN-τ for 6 h, and 5 μg/mL LPS for 12 h adding 1 μM TAK242 for 1.5 h before adding LPS. (**A**–**C**): The protein levels of TLR4, p-ERK, and ERK were determined using Western blot; (**D**,**E**): The mRNA levels of Th1 cytokines (*IL-1β*, *IL-8*, *TNF-a*, and *IL-6*) and the mRNA levels of Th2 cytokines (*IL-10* and *IL-4*) were determined using qRT-PCR; (**F**): The mRNA levels of, *ITGB3*, *ITGB5*, *VEGF*, and *LIF* were determined using qRT-PCR; (**G**–**I**): The protein expression of Th1 pro-inflammatory cytokines (TNF-a and IL-6) and Th2 anti-inflammatory cytokines (IL-10 and IL-4) were detected by Western blot.; Data are represented as the mean ± SEM of three independent experiments. *, *p* < 0.05; **, *p* < 0.01.

**Figure 6:**
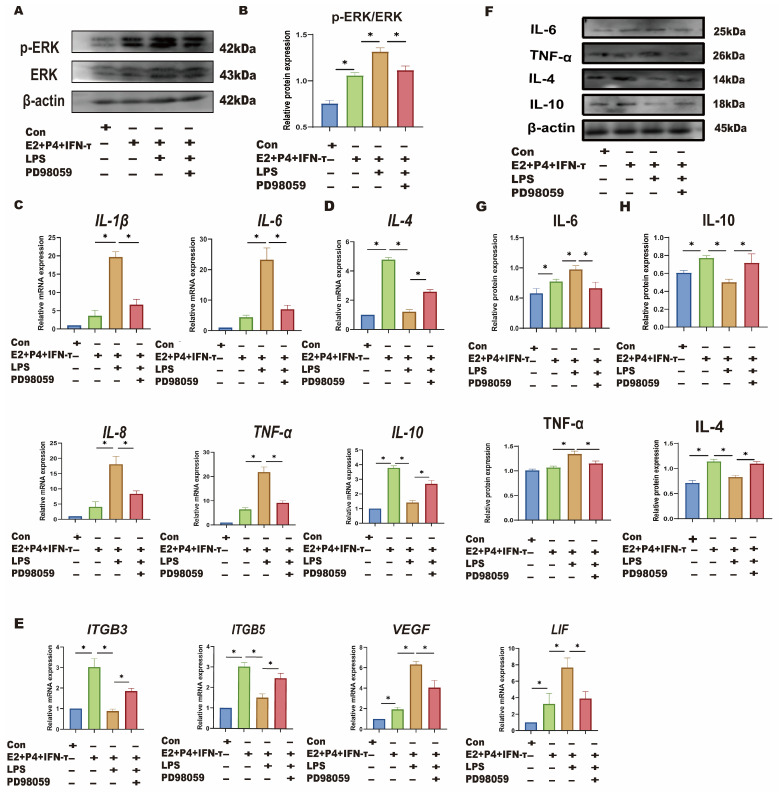
LPS affects endometrial receptivity in sheep through the TLR4/ERK pathway. The sEECs were treated with 10^−7^ M P_4_, 10^−9^ M E_2_ for 12 h, 20 ng/mL IFN-τ for 6 h, and 5 μg/mL LPS for 12 h, adding 10 μM PD98059 before using LPS for 1.5 h. (**A**,**B**): The protein levels of p-ERK and ERK wwere determined using Western blot; (**C**,**D**): The mRNA levels of Th1 cytokines (*IL-1β*, *IL-8*, *TNF-a*, and *IL-6*) and the mRNA levels of Th2 cytokines (*IL-10* and *IL-4*) were determined using qRT-PCR; (**E**): The mRNA levels of *ITGB3*, *ITGB5*, *VEGF*, and *LIF* were determined using qRT-PCR; (**F**–**H**): The protein expression of Th1 pro-inflammatory cytokines (TNF-a and IL-6) and Th2 anti-inflammatory cytokines (IL-10 and IL-4) were detected by Western blot. Data are represented as the mean ± SEM of three independent experiments. *, *p* < 0.05.

sEECs were treated with TLR4 and ERK inhibitors to investigate whether LPS affects the sheep endometrium through the TLR4/ERK pathway. sEECs were treated with E_2_, P_4_, and IFN-τ to mimic endometrial receptivity in vitro. As shown in Figure 5A–C, LPS treatment in the endometrial receptivity model significantly increased TLR4 and p-ERK expression levels (*p* < 0.05). TLR4 inhibitor TAK242 treatment significantly inhibited the TRL4 expression level and p-ERK/ERK ratio compared with LPS treatment (*p* < 0.05). qRT-PCR and Western blot were used to detect the changes in Th1-Th2 cytokines (Figure 5D,E,G,H) and implantation genes (Figure 5F). The results showed that LPS treatment significantly increased Th1 cytokines (TNF-a, IL-8, IL-6, and IL-1β) and decreased the Th2 cytokines (IL-10 and IL-4), suggesting that LPS also causes Th1 excursions in sEECs. Moreover, the expression levels of the attachment genes (*VEGF* and *LIF*) were significantly increased, while those of the adhesion genes (*ITGB3* and *ITGB5*) were significantly decreased (Figure 5F), indicating that LPS also led to disordered expression of genes relative to embryo implantation. After TLR4 inhibitor treatment, the expression levels of Th1 cytokines (TNF-a, IL-8, IL-6, and IL-1β) and the attachment genes (*VEGF* and *LIF*) significantly decreased, and the expression levels of Th2 cytokines (IL-10 and IL-4) and the adhesion genes (*ITGB3* and *ITGB5*) significantly increased. The addition of ERK inhibitor PD98059 significantly reduced phosphorylation of ERK caused by LPS in endometrial receptivity mode (*p* < 0.05; Figure 6A,B). The addition of the ERK inhibitor PD98059 resulted in similar results [Th1, Th2 cytokines, the adhesion genes *(ITGB3* and *ITGB5*), and the attachment genes (*VEGF* and *LIF*)] to the addition of the TLR4 inhibitor (*p* < 0.05; Figure 6C–H).

### 3.6. PTE Alleviates LPS-Induced sEECs Injury

**Figure 7:**
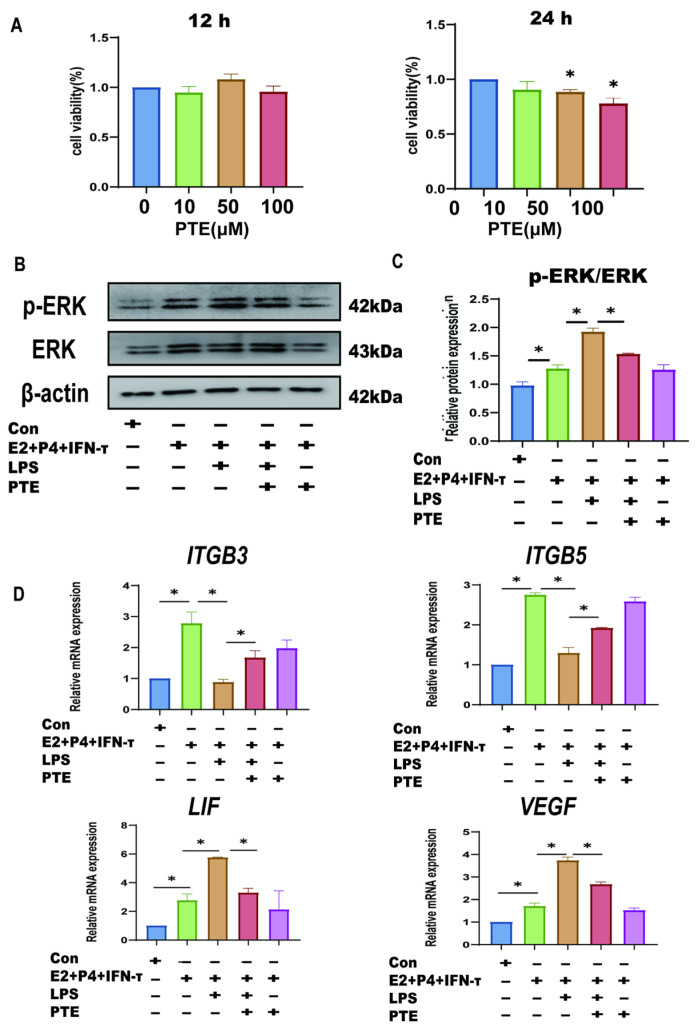
Effect of pterostilbene on endometrial epithelial cells of sheep under LPS. (**A**): The cell viability of sEECs treated with 10, 50, and 100 μM PTE for 12 h and 24 h by the CCK-8 assay. (**B**,**C**): The protein levels of p-ERK and ERK were determined using Western blot. (**D**): The mRNA levels of *ITGB3*, *ITGB5*, *VEGF,* and *LIF* were determined using qRT-PCR; Data are represented as the mean ± SEM of three independent experiments. *, *p* < 0.05.

Based on the above studies, we have elucidated that LPS disrupts the immune mi-croenvironment of the sheep endometrium by activating the TLR4/ERK pathway, which leads to the disruption of embryo attachment gene expression. However, in recent years, many articles have reported that PTE has an inhibitory effect on LPS-induced inflammation and oxidative damage [28,29,30]. The activation of ERK and other inflammatory pathways in the presence of LPS can cause damage to cells, and the inhibitory effect of PTE on the ERK signaling pathway has been verified in many tissues and epithelial cells [31,32]. As a naturally existing substance, PTE has a certain safety and tolerability [31]. We hypothesized that PTE could alleviate the damaging effect of LPS on the sheep endometrium by inhibiting the ERK pathway. Cell viability was examined to assess the toxicity of PTE in LPS-induced sEECs. The administration of sEECs to 10, 50, and 100 μM PTE for 12 h did not affect cell viability (Figure 7A). When exposed to 50 and 100 μM for 24 h, the viability of sEECs was significantly affected. Therefore, the suitable concentrations of 50 μM PTE for 12 h were used for the consequent analysis to omit the probability that the suppressive result of PTE on LPS-induced damage was an effect of cytotoxicity triggered by cell viability reduction. p-ERK expression was significantly exhibited and the p-ERK/ERK ratio significantly increased after the addition of LPS. After co-treatment with LPS and PTE, p-ERK expression was significantly inhibited, and the p-ERK/ERK ratio significantly decreased after the addition of LPS (Figure 7B,C). qRT-PCR was used to detect changes in the implantation-related genes. The results showed that attachment gene (*VEGF* and *LIF*) expression significantly increased, and that of adhesion genes (*ITGB3* and *ITGB5*) significantly decreased after the addition of LPS (Figure 7D). Compared with the LPS group, co-treatment with LPS and PTE significantly decreased the expression of the attachment genes (*VEGF* and *LIF*) and significantly increased that of adhesion genes (*ITGB3* and *ITGB5*).

## 4. Discussion

In this study, we investigated the damage of LPS to the endometrium of sheep from the perspective of immunity and implantation-related genes expression based on the TLR4/ERK pathway.

To date, many studies on mice and humans have shown that during pregnancy, the maternal immune system undergoes changes to develop immune tolerance towards the semi-allogeneic fetus, preventing immune rejection responses [32]. In the present study, the expression of Th1 cytokines (except IL-1β) increased from day 0 to day 16 and decreased from day 16 to day 20. The expression of Th2 cytokines continued to increase throughout implantation, suggesting that normal pregnancy appears to occur in the presence of enhanced Th2 cytokines. The results of the present study are in agreement with many reports from previous studies on mouse, sheep, and cow pregnancies [33,34,35]. The upward trend of IL-1β throughout the implantation period of the embryo does not seem to fit this conclusion. IL-1β expression in the pig conceptus rapidly increases during the short elongation period and then (about day 70) decreases dramatically (2000 times) as the conceptus attaches to the uterine surface [36]. However, for pigs, the conceptus’s production of IL-1β temporally increases during the period of trophoblast remodeling during elongation in the first 14 days, similar to the sheep endometrial epithelium at day 12. The most significant pig placental remodeling of the pregnancy takes place between 60 and 70 days [37]. Similar to the sheep endometrial epithelium at day 20, IL-1β may collaborate to promote implantation [37]. It has been suggested that IL-1β is necessary for promoting early conceptus development and rapid elongation, enhancing uterine receptivity for implantation, and increasing the permeability of endometrial blood vessels, thereby facilitating fetal–maternal hemotrophic exchange [38]. Szostek et al. revealed that the up-regulation in TNF-α mRNA expression might be implicated towards increased PGE_2_ production (luteotrophic) and reduced PGF2α secretion (luteolytic) [39]. In this study, there was a significant increase in Th1 cytokines and a significant decrease in Th2 cytokines after LPS infusion on days 12, 16, and 20 of pregnancy, suggesting that LPS infusion can lead to endometrial Th1 deviation. Excessive Th1 cytokines in early pregnancy can lead to adverse pregnancy and embryo implantation failure [40]. However, in studies of LPS-induced endometritis in cattle, *E*. *coli* increased the expression of Th2 factor IL-10 [41]. The signaling pathways in the body are complex, especially during the special period of pregnancy when the embryo is implanted. IFNT, ruminant pregnancy recognition protein, induces the expression of IFN-stimulated genes (ISGs) in bovine neutrophils through Janus kinase (JAK) 3 and phosphoinositide 3-kinase (PI3K), which lead to increased production of interleukin IL-10 [42]. LPS inhibited the activation of JAK/STAT signaling pathway during the implantation of sheep embryos after artificial insemination [27], resulting in a decrease in IL-10.

ITGB3 and ITGB5 were classical adhesion genes [43]. Increased expression in adhesion proteins are closely related to the formation of endometrial receptivity [44]. ITGB3 appears in the luminal and glandular EECs at the putative time of implantation in humans [45]. Decreased expression of ITGB3 in the endometrium of humans at the mRNA and protein levels reduced the adhesion and invasion ability [46]. In this study, the expression of ITGB3 and ITGB5 decreased significantly on days 12, 16, and 20 after LPS infusion, suggesting that LPS infusion inhibited adhesion. In the endometrium, LIF is expressed in a cycle-dependent manner, with the highest level occurring at the time of implantation. In sheep, the highest LIF expression by the endometrium was observed at days 16–20 of gestation [47]. LIF enhances the adhesion of trophoblastic cells to endometrial cells by up-regulating the expression of integrin heterodimer αVβ3 and αVβ5 [48]. Female LIF knockout mice were fertile, but blastocysts failed to implant [49]. Our results showed that LIF levels were significantly higher on days 16 and 20 of normal pregnancy than on days 0 whereas LPS infusion on days 16 and 20 resulted in a significant increase in LIF. This result is similar to the study conducted by Marina Izvolskaia, who showed that a single administration of low LPS doses (45 µg/kg) to mice on gestation day 11.5 considerably increased the levels of LIF in the maternal and fetal serum and amniotic fluid after 1.5 h [50]. This suggests that LIF not only regulates the attachment process but also participates in the inflammatory response. LIF promotes the expression of TLR2 and TLR4 receptors and their corresponding ligands Myd88 and CD14 in LE and matrix. The increase in TLR2 expression may be tightly regulated, as TLR2/6 activation leads to a decrease in the implantation rate by increasing the expression of interleukin (IL-1β) and monocyte chemoattractant protein (MCP)-1 [51]. This suggests that LIF not only regulates the attachment process but also participates in the inflammatory response. During the process of animal implantation, the number of endometrial blood vessels increased with the number of days, and promoting angiogenesis can enhance endometrial receptivity, with VEGF being a key factor in this process [52]. In the endometrium of pigs, the mRNA expression levels of VEGF and its receptors were higher than those of the estrous cycle on the 18th day of gestation and peaked during the gestation cycle [53]. We found that VEGF levels were significantly elevated on day 12, day 16, and day 20 in normal pregnancy, while after LPS infusion on days 12, 16, and 20, the expression of VEGF still increased. It possibly causes inflammation, as VEGF increases vascular permeability, leading to extra vasation of plasma proteins, fluid accumulation, and oedema, which contribute to an increase in the extent of inflammation [54]. Disturbances in uterine blood supply are associated with higher perinatal morbidity and mortality [55]. VEGF increase has also been implicated in rejection, leading to endometrial lesions and affecting the receptivity of the endometrium [56]. The maternal VEGF increase results with abortion due to endothelial dysfunction [57].

As a specific receptor for recognizing LPS, TLR4 serves as the core hub connecting LPS and the balance of pregnancy immune tolerance and plays a crucial role in the establishment and maintenance of pregnancy. Early pregnancy causes an up-regulation of the TLR4 gene and protein in the cervical epithelium, which is involved in the regulation of the innate immune response caused by hormonal secretion in humans [58]. Meanwhile, TLR4 is an important cellular signaling pathway regulating inflammatory and immune responses [59]. TLR4 expression is enhanced in the thymus of Swiss albino mice with experimental endotoxemia [60]. TLR4 expression was higher on days 0 and 16 in leukocytes collected from heifers that lost embryos on day 60 compared to pregnant heifers that maintained pregnancy [61]. Concentrations of TNF-a and NO in the serum of aborted goats were significantly higher than during pregnancy [62]. The ERK signaling pathway might be involved in trophoblast modification invading the endometrium, whereas the abnormal signaling pathway might cause spontaneous pregnancy loss [63]. Abnormal expression of the ERK pathway affects not only the normal development of trophoblast cells but also the normal physiological function of the endometrium. Abnormal activation of the ERK pathway, impaired expression of decidua-related genes, and inactivation of Src can cause 8-Br-c AMP to impair endometrial decidualization, which leads to embryo implantation failure or abortion [64]. To determine the molecular mechanism by which LPS affects endometrial receptivity in sheep via TLR4/ERK, we assessed the protein levels of the TLR4/ERK signaling pathway. The results showed a significant increase in the expression of TLR4 and p-ERK proteins on days 12, 16, and 20 of normal pregnancy compared with day 0. The expression of TLR4 and p-ERK proteins continued to be significantly up-regulated after LPS infection at these three stages compared to normal pregnancy. The results suggest that LPS abnormally activates the TLR4/ERK pathway at days 12, 16, and 20. To validate the effect of LPS on endometrial receptivity in sheep, we established in vitro models of inflammation and receptive state of sheep endometrial epithelial cells. We determined the optimal concentration of 5 μg/mL LPS in vitro. At the same time, we found that LPS caused G1 phase arrest of the sEEC cell cycle. In bovine endometrial stromal cells, LPS increased apoptosis, hindered cell cycle progression by blocking it in the G1 phase [65]. After LPS activated the TLR4 receptor, the MAPK pathway could regulate the cell cycle of macrophages. It may be considered a key factor for the analysis of the inflammatory response [66]. Additionally, cell cycle arrest is an important cellular process for preventing the death of epithelial cells caused by bacteria or disease, especially in the early stages, by limiting pro-apoptotic triggers, inhibiting cell cycling and DNA replication, and maintaining energy homeostasis and cellular organelle function [67].

The results showed that TAK424, a TLR4 inhibitor, significantly down-regulated Th1 cytokines, up-regulated Th2 cytokines, and alleviated LPS-induced aberrant expression of implantation genes and persistent elevation of TLR4 compared with the LPS group. We also found that the TLR4 inhibitor also reduced the phosphorylation level of ERK, suggesting that LPS may have a damaging effect on endometrial cells through the TLR4 downstream pathway protein ERK. Subsequently, we treated sEECs with an inhibitor of ERK phosphorylation, and the results were consistent with the TLR4 inhibitor. Taken together, LPS leads to impaired endometrial receptivity through the TLR4/ERK signaling pathway.

PTE, a naturally dimethylated analogue of resveratrol, has been shown to have anti-inflammatory and anticancer effects. PTE mitigates LPS-induced lung injury through activating NR4A1 [68], ameliorates LPS/D-Gal-induced liver injury by inhibiting nuclear factor-kappa B (NF-κB) and up-regulating Nrf2 and heme oxygenase-1 (HO-1), and interferes with LPS-induced myocardial injury through oxidative stress and inflammasome pathways as well as protects against LPS-induced blood–brain barrier damage in immortalized brain endothelial cells in vitro. However, the role of PTE on LPS-induced endometrial injury and the detailed mechanisms underlying these activities remain unclear. Here, using LPS and an in vitro endometrial receptivity model, we found by WB and q-PCR that LPS could abnormally activate the p-ERK/ERK ratio in the receptivity model, and the addition of PTE significantly reduced the LPS-induced increase in the p-ERK/ERK ratio, and PTE treatment also alleviated LPS-induced damage to the attachment genes (VEGF and LIF) and adhesion genes (ITGB3 and ITGB5) induced by LPS; thus, PTE treatment significantly alleviated LPS-induced endometrial damage, and the mechanism may inhibit the expression of the ERK signaling pathway. Similarly, in a study of the anticancer effects of PTE, it was shown that PTE could inhibit the EMT of triple-negative breast cancer cell lines through the ERK pathway, thus exhibiting anticancer effects [69]. PTE has the potential to treat ovarian cancer by reducing the level of TNF-a cytokine via inhibition of AKT- and ERK-mediated pathways in the human ovarian cancer cell line, IGROV-1 cells [70]. The highest therapeutic potential of pterostilbene is by effectively targeting cell death determinants in endometriosis [71].

Overall, LPS infusion on days 12, 16, and 20 of pregnancy impaired endometrial receptivity and immunological tolerance in sheep through the TLR4/ERK pathway, and PTE can alleviate the endometrial damage caused by LPS via the ERK pathway, which may have important implications for mechanism study and treatment of embryo implantation failure. This study advances the understanding of pregnancy immune tolerance mechanisms in non-invasive embryo implantation species and elucidates the potential molecular pathways through which LPS disrupts cellular and molecular signaling, regulating uterine receptivity and implantation.

## 5. Conclusions

**Figure 8:**
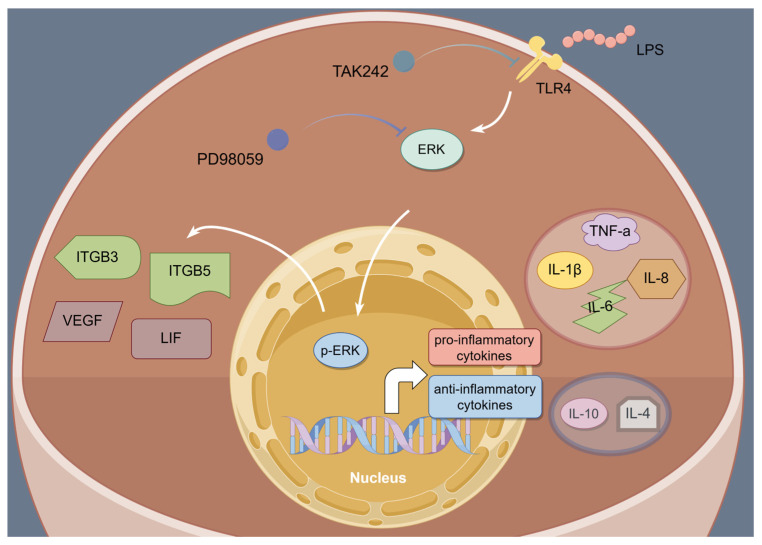
Schematic figure depicting the potential role of LPS on endometrial receptivity in sheep. In the presence of LPS, the TLR 4/ERK signaling pathway is activated, and TLR4 and ERK phosphorylation inhibitors significantly reduced the expression of TLR4 and p-ERK, down-regulated Th1 cytokines, up-regulated Th2 cytokines, and alleviated the disruption of genes for attachment.

In summary, this study clarified that LPS infusion during the implantation of sheep embryos interferes with the balance of Th1–Th2 in the endometrial microenvironment and disrupts the expression of implantation-related genes by overactivating TLR4/ERK signals (Figure 8). In addition, PTE can alleviate the cell damage caused by LPS. These findings offer new and potential insights into the mechanisms by which LPS impacts endometrial receptivity and provide potential therapeutic targets for the prevention and treatment of implantation failure associated with immune imbalance.

## Figures and Tables

**Table 1:** Primer sequences for genetic testing.

Gene	Accession Number	Forward Primer Sequence (5′-3′)	Tm	Reverse Primer Sequence (5′-3′)	Tm	Product Length (bp)
*IL-1* *β*	NM_001009465.2	F: AGCCGAGAAGTGGTGTTCTG	59.97	R:TGGCCACCTCTAAAACGTCC	59.96	314
*TNF-α*	NM_001024860.1	F:AACAGGCCTCTGGTTCAGACA	61.32	R: CCATGAGGGCATTGGCATAC	59.04	133
*IL-6*	NM_001009392.1	F: GACACCACCCCAAGCAGACTA	61.72	R: TGCCAGTGTCTCCTTGCTGTT	62.2	144
*IL-4*	NM_001009313.3	F: ATCATCGGCATTTTGAACGAGG	60.23	R: TGCAGCTCCATGAGAACACTA	61.67	129
*IL-8*	NM_001009401.2	F: TCCTGCTCTCTGCAGCTCTGT	63.24	R: GGGTGGAAAGGTGTGGAATG	58.74	100
*IL-10*	NM_001009327.1	F: TGCTGGATGACTTTAAGGG	56.97	R: AGGGCAGAAAACGATGACA	59.34	184
*ITGB3*	XM_060395753.1	F: GGTGGTCTTGCTATCCGTGA	59.46	R: GTTGTTGGCAGTGTCCCATG	59.96	151
*ITGB5*	XM_027956587.3	F:GGGTCCGATGTCATCCAGC	60.23	R: GAACGTAGTCTTGTCACCGGG	60.76	72
*LIF*	XM_012098046.5	F:TATCGCATCATCGCGTACCT	59.92	R: ACATGGCTCACGTGGTACTT	59.31	176
*VEGF*	NM_001025110.1	F: CATGGATGTCTACCAGCGCA	60.18	R: TGGGCACACACTCCAGACTT	61.34	161
*GAPDH*	NM_001190390.1	F: CAAGTTCCACGGCACAGTCA	60.80	R: TGGTTCACGCCCATCACAA	59.85	249

## Data Availability

The data presented in this study are available upon request from the corresponding author. The availability of the data is restricted to investigators based in academic institutions.

## Supplementary Materials

The following supporting information can be downloaded at: https://www.mdpi.com/article/10.3390/ani15121712/s1, Figure S1: original Western Blot figures of Figure 1E; Figure S2: original Western Blot figures of Figure 1H; Figure S3: original Western Blot figures of Figure 3A; Figure S4: original Western Blot figures of Figure 3D; Figure S5: original Western Blot figures of Figure 5A and G; Figure S6: original Western Blot figures of Figure 6A and F; Figure S7: original Western Blot figures of Figure 7B.
