# Supplementary material for: LPS Regulates Endometrial Immune Homeostasis and Receptivity Through the TLR4/ERK Pathway in Sheep
Source: Animals (Basel). 2025 Jun 10;15(12):1712. doi: 10.3390/ani15121712 (PMC12189910; doi:10.3390/ani15121712)

Figure S1: original Western Blot figures of figure 1E

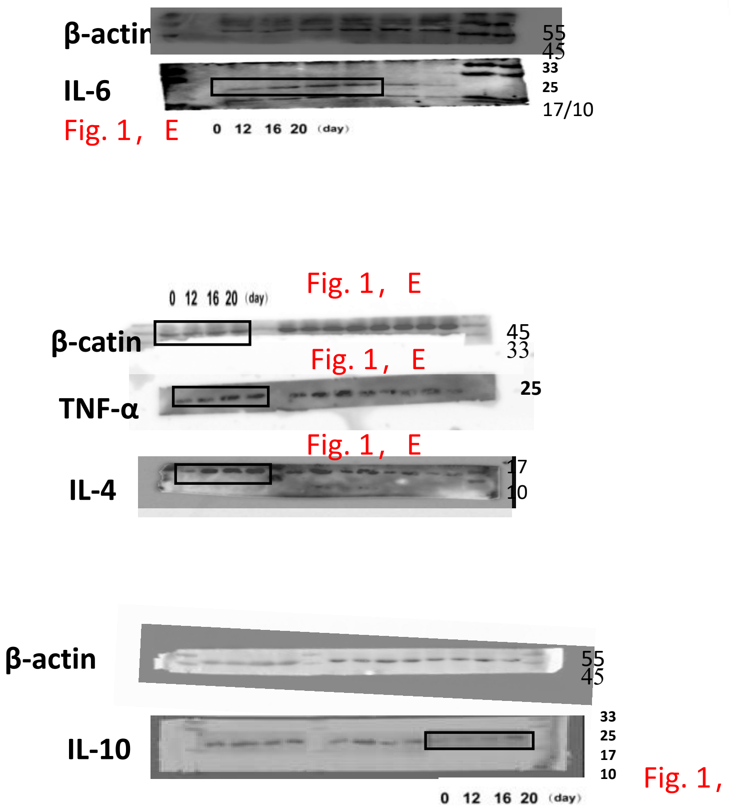

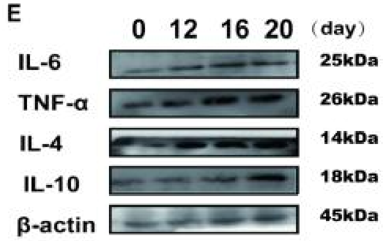

all molecular weight markers

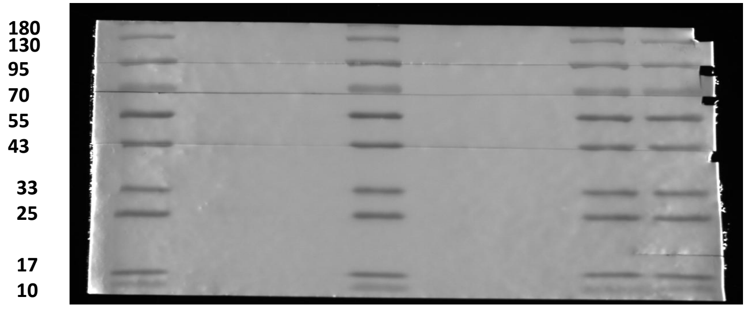

Figure S2: original Western Blot figures of figure 1H

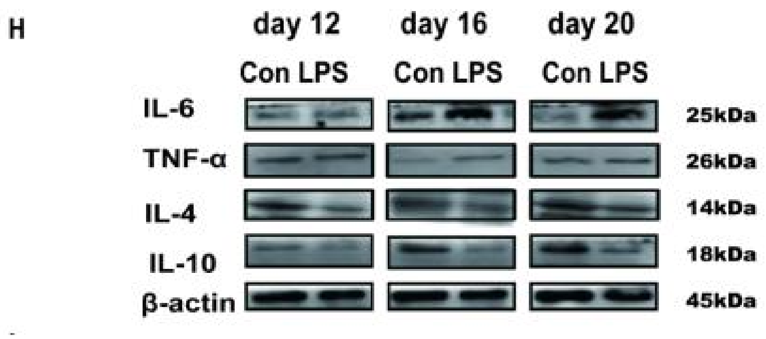

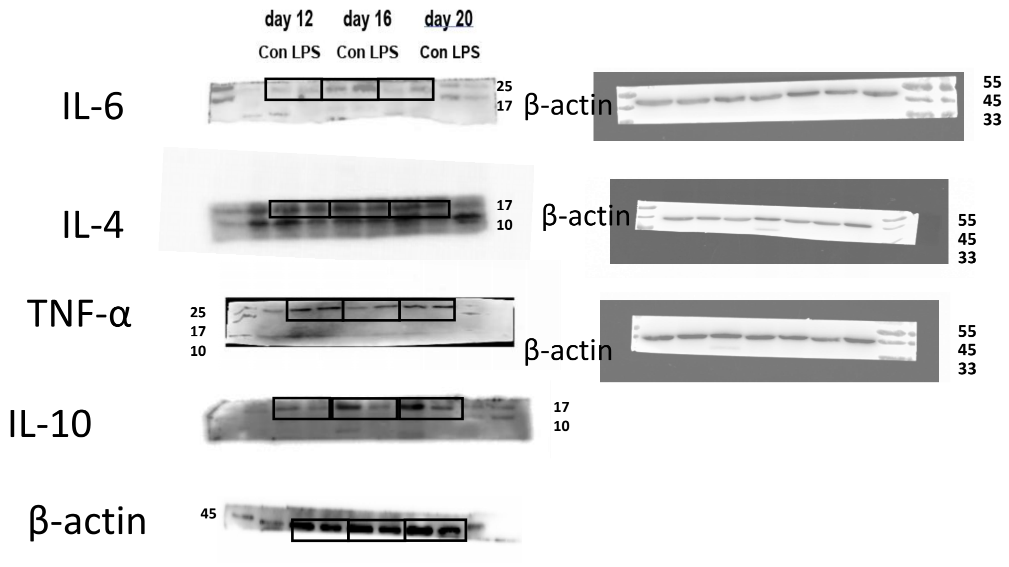

Figure S3: original Western Blot figures of figure 3A

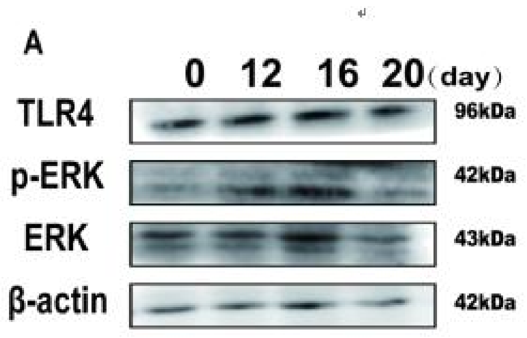

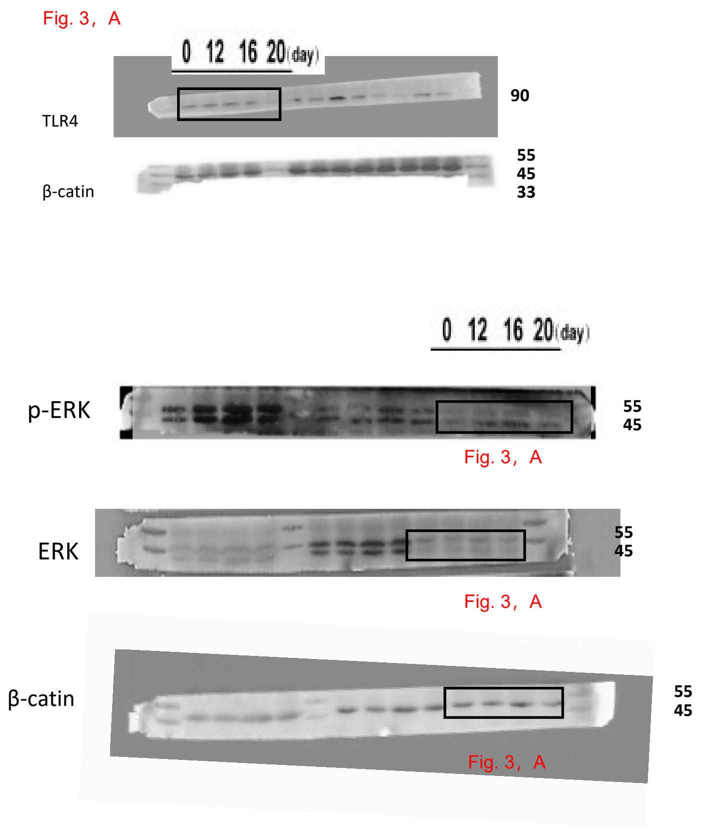

Figure S4: original Western Blot figures of figure 3D

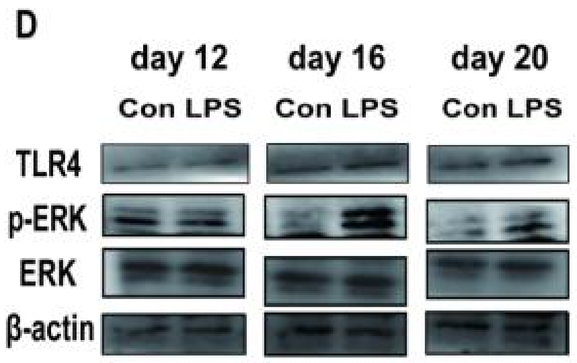

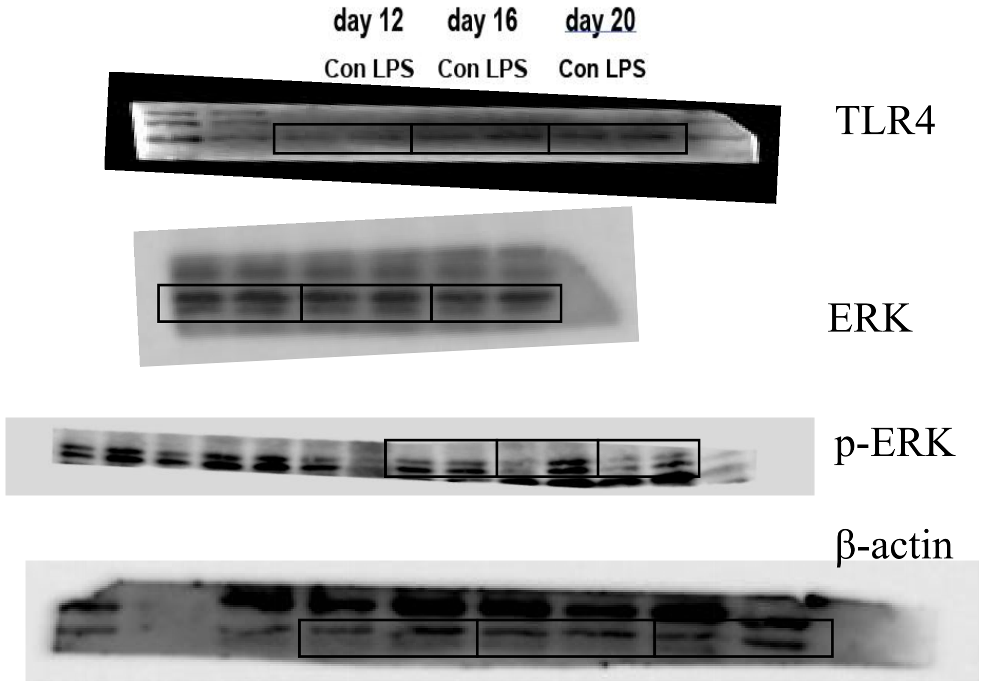

Figure S5: original Western Blot figures of figure 5A and G

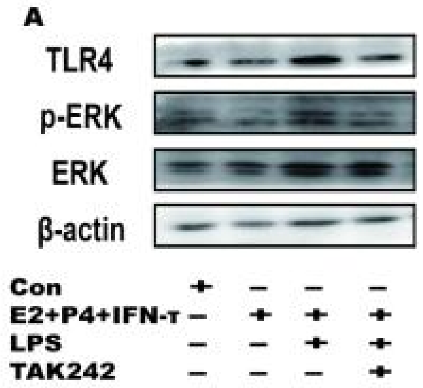

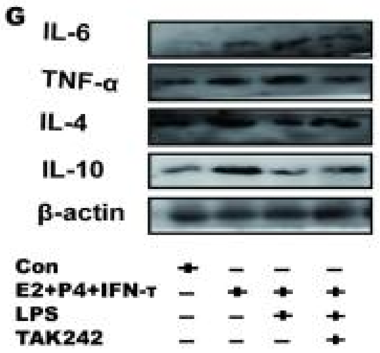

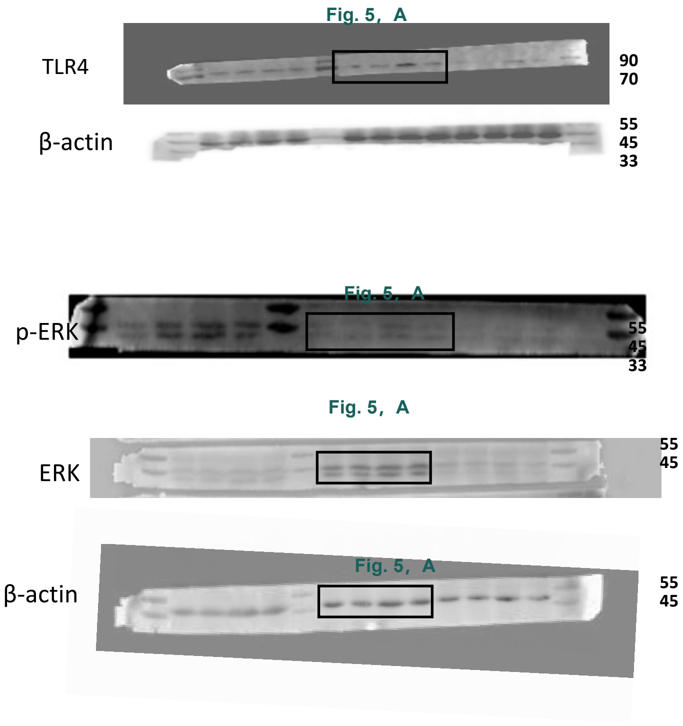

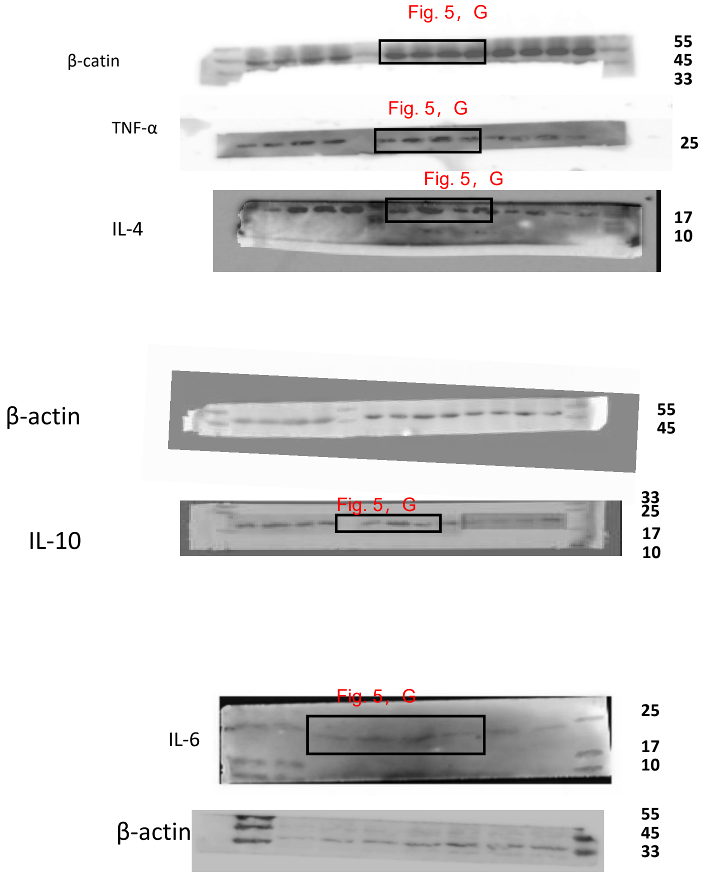

Figure S6: original Western Blot figures of figure 6A and F

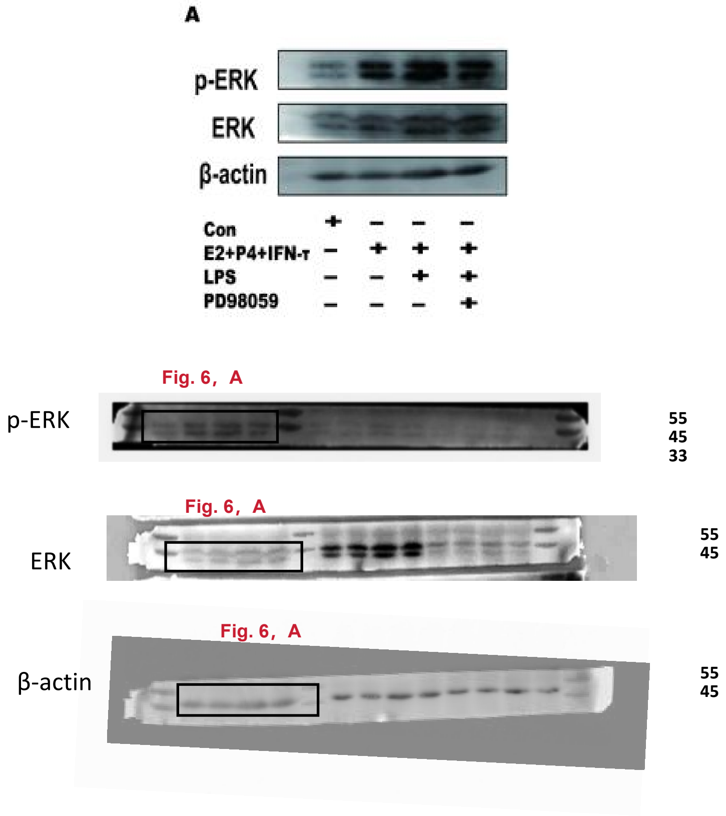

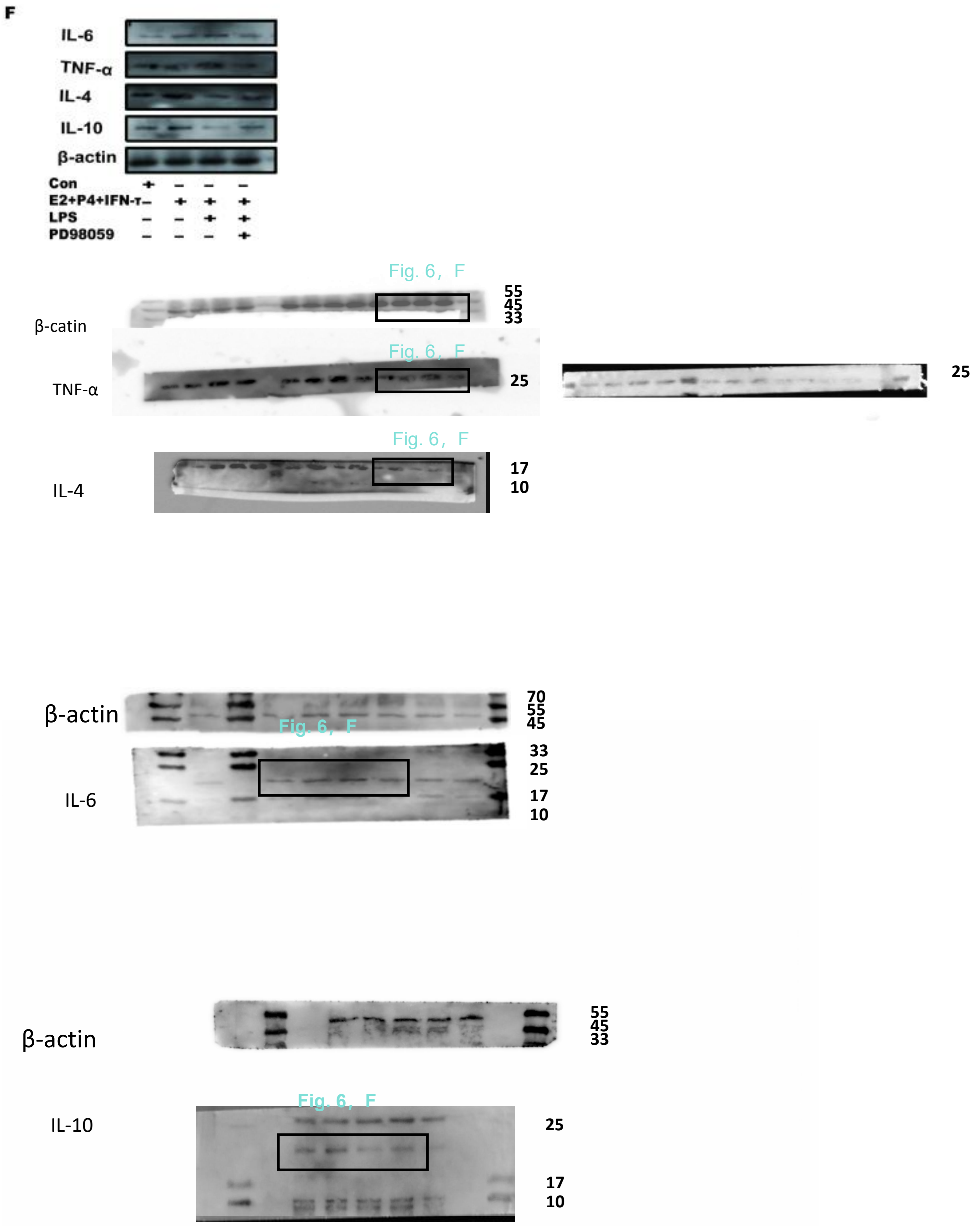

Figure S7: original Western Blot figures of figure 7B

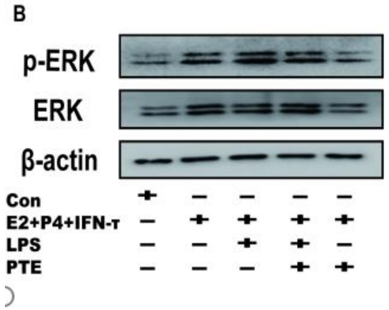

ERK

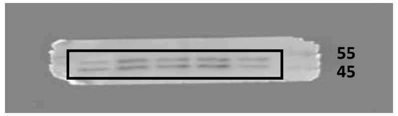

β-actin

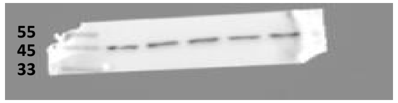

p-ERK

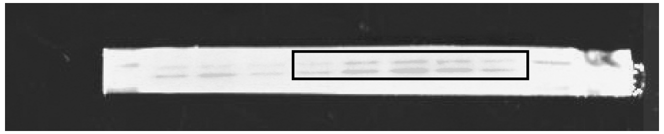

β-actin

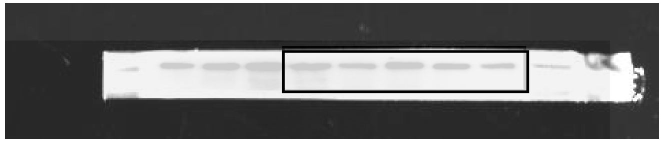

Supplement: Supplementary file 1 [file animals-15-01712-s001.zip › animals-3545748-supplementary.pdf]
